# Bioinformatics and Biomedical Informatics

**DOI:** 10.1155/2013/591976

**Published:** 2013-05-29

**Authors:** Kayvan Najarian, Rachid Deriche, Mark A. Kon, Nina S. T. Hirata

**Affiliations:** ^1^Department of Computer Science, Virginia Commonwealth University, 401 West Main Street, P.O. Box 843019, Richmond, VA 23284, USA; ^2^Inria Sophia Antipolis-Méditerranée Research Center, 2004 Route des Lucioles, BP 93, 06902 Sophia Antipolis Cedex, France; ^3^Department of Mathematics and Statistics, Boston University, 111 Cummington Street, Boston, MA 02215, USA; ^4^Department of Computer Science, Institute of Mathematics and Statistics, Rua do Matão, 1010 05508-090 São Paulo, SP, Brazil

New and novel high-throughput systems for measurement of biological and physiological data have created a challenge: processing and analyzing the abundant resulting datastreams. There is a need for advanced computational methods to process these often very large datasets, and more generally to form decision-support systems for complex problems in medicine, biology, and related fields. During the last few decades, the vital role of these computational methods, in particular in emerging fields of life sciences such as translational medicine, has been further recognized. The need for more efficient computational methods has highlighted their roles as fundamental elements of almost any endeavor in today's life sciences. The current focus of these fields is on designing computational methods that facilitate the design and development of systems for clinical applications.

This special issue serves as a brief update to the current status of and advances in methods of biomedical informatics and bioinformatics. The computational approaches presented span a number of methods, from newly developed algorithms in DNA microarray analysis to biomedical signal/image processing techniques. The particular diseases as well as remedies addressed, from novel components of schizophrenia to new diagnoses of breast cancer, address major health issues that any society today is likely to be facing. 

The paper by M. Logotheti et al. provides a comparative genomic study in patients suffering from schizophrenic and bipolar disorders using microarray expression profiling meta-analysis. A. Belle et al. present a survey of biomedical informatics, methods, and applications, as applied to computer-aided decision support systems. The paper by J. H. Phan et al. presents a meta-analysis-based feature selection method to combine multiple microarray datasets and improve reproducibility in identification of informative genes and subsequent clinical prediction. Finally, M. Burton et al. provide a cross-study comparison of classification methods of gene expression profiles for predicting metastasis in breast cancer. 

With the current burst of new biomedical measurement systems (in particular portable monitoring devices) among many new data sources, there are now petabyte and higher capacity biomedical databases requiring processing via ever-more efficient computational methods. Work on their designs in bioinformatics and biomedical informatics will accelerate further their quality and capacity, as imaging and monitoring technologies produce ever larger quantities of more detailed and more informative data. These works are continuing the advance of the above technologies and their frontiers.

*Kayvan Najarian* *Kayvan Najarian* *Rachid Deriche* *Rachid Deriche* *Mark A. Kon* *Mark A. Kon* *Nina S. T. Hirata* *Nina S. T. Hirata*

